# Empoisonnement criminel au Maroc: données du Centre Antipoison et de Pharmacovigilance du Maroc, 1980-2014

**DOI:** 10.11604/pamj.2021.38.42.27450

**Published:** 2021-01-15

**Authors:** Sara Boukhorb, Naima Rhalem, Soumaia Hmimou, Abdelmajid Soulaymani, Abdelrhani Mokhtari, Rachida Soulaymani-Bencheikh, Rachid Hmimou, Hinde Hami

**Affiliations:** 1 Faculté des Sciences, Université Ibn Tofail, Kénitra, Maroc,; 2 Centre Antipoison et de Pharmacovigilance du Maroc, Rabat, Maroc,; 3 Faculté de Médecine et de Pharmacie, Université Mohammed V, Rabat, Maroc

**Keywords:** Empoisonnement criminel, intoxication intentionnelle, CAPM, Maroc, Criminal poisoning, intentional intoxication, MPCPC, Morocco

## Abstract

**Introduction:**

les intoxications intentionnelles constituent un problème majeur de santé publique, aussi bien dans les pays développés que dans les pays en développement. L´objectif de la présente étude est de décrire le profil épidémiologique des intoxications criminelles au Maroc.

**Méthodes:**

il s'agit d'une étude rétrospective de tous les cas d'intoxications criminelles recensés par le Centre Antipoison et de Pharmacovigilance du Maroc (CAPM) entre 1980 et 2014.

**Résultats:**

durant la période d'étude, 611 cas d'empoisonnements criminels ont été enregistrés, soit 2,1% de l'ensemble des intoxications intentionnelles déclarées durant la même période. L'âge moyen des intoxiqués était de 26,4±14,3 ans. Plus d'un quart des cas étaient des enfants de moins de 15 ans (28,6%). D'après les résultats de l'étude, 55,9% étaient de sexe masculin, avec un sex-ratio (M/F) de 1,3. La majorité des cas, soit 89,4% étaient survenus en milieu urbain. Les intoxications ont été collectives dans 24,4% des cas. Les produits les plus fréquemment utilisés étaient les pesticides (19,1%) et les plantes (19%). Les signes présentés étaient variables suivant le ou les toxiques en cause, la quantité ingérée et le délai écoulé avant le traitement. Toute une gamme de troubles digestifs, neurologiques, respiratoires et cardio-vasculaires a été retrouvée. Parmi les 440 cas pour lesquels on disposait de données sur l´évolution, 27 sont décédés. Les autres cas ont survécu avec ou sans séquelles.

**Conclusion:**

l'empoisonnement criminel reste un problème non négligeable. Le nombre de cas est probablement sous-estimé du fait du nombre important de cas non diagnostiqués ou non déclarés.

## Abstract (en)

**Introduction:**

intentional poisoning is a major public health problem in both developed and developing countries. The purpose of this study is to describe the epidemiological features of criminal intoxication in Morocco.

**Method:**

we conducted a retrospective study of all cases of criminal intoxication identified by the Morocco Poison Control and Pharmacovigilance Centre (MPCPC) between 1980 and 2014.

**Results:**

during the study period, 611 cases of criminal poisoning were recorded, reflecting a rate of 2.1% of all intentional poisoning reported during the same period. The average age of intoxicated patients was 26.4±14.3 years. More than a quarter of the subjects were children under the age of 15 (28.6%). According to the study results, 55.9% were male, with a sex-ratio (M/F) of 1.3. The majority of cases (89.4%) occurred in urban areas. Collective intoxications were reported in 24.4% of cases. The most frequently used products were pesticides (19.1%) and plants (19%). Patients developed different symptoms based on the toxic substances used, the amount ingested and the time elapsed before treatment. A range of digestive, neurological, respiratory and cardiovascular disorders were reported. Out of 440 patients with outcome data available, 27 died. The remainder of patients survived with or without sequelae.

**Conclusion:**

criminal poisoning is a major issue. The number of cases is probably underestimated due to a large number of undiagnosed or unreported cases.

## Introduction

L´Organisation Mondiale de la Santé (OMS) a estimé que 25 à 33% de la charge mondiale de morbidité est attribuable à des risques toxiques et 3% des hospitalisations sont dues aux intoxications [1,2]. Les intoxications intentionnelles restent un problème majeur de santé publique, aussi bien dans les pays développés que dans les pays en développement. Parmi elles, les actes criminels ne sont pas négligeables. Selon les statistiques du Centre Suisse d´Information Toxicologique, 35948 cas d´intoxications ont été enregistrés en Suisse en 2018 dont 107 cas en rapport avec des actes criminels [3].

Au Maroc, les intoxications intentionnelles représentent 15,75% de l´ensemble des intoxications déclarées au Centre Antipoison et de Pharmacovigilance du Maroc (CAPM) en 2014 [4]. Les circonstances criminelles sont rarement étudiées dans la littérature et les causes restent généralement inconnues. L´administration de substances toxiques à l´insu de la victime à des fins criminelles peut causer des effets graves, voire mortels. L´objectif de ce travail était de décrire le profil épidémiologique des intoxications criminelles au Maroc durant la période 1980-2014.

## Méthodes

Il s´agit d´une étude rétrospective des cas d´intoxications criminelles signalés au CAPM sur une période de 35 ans allant de janvier 1980 jusqu´à décembre 2014. Les données ont été collectées à partir des fiches de déclaration des intoxications reçues à l'unité de toxicovigilance et des appels reçus par l´unité de l´information toxicologique.

Les principales informations collectées étaient: le sexe, l´âge, le milieu de résidence, le lieu d´intoxication, le produit en cause, les signes cliniques et symptômes et l´évolution clinique des patients intoxiqués. L´âge des patients est présenté selon la classification de Programme International sur la Sécurité des Substances Chimiques (l´IPCS): enfant «5-14 ans», adolescent «15-19 ans», adulte «20-74 ans», personne âgée «75 ans ou plus».

L´évaluation de la gradation est faite selon «Poisoning Severity Score (PSS)»: grade 0 (néant), grade 1 (mineur), grade 2 (modéré), grade 3 (sévère), grade 4 (fatal) [5]. Les indicateurs de santé liés aux intoxications criminelles ont été calculés suivant les projections de population publiées par le Haut-Commissariat au Plan (HCP) du Maroc.

Une intoxication criminelle est définie comme étant une exposition ou incident où des patients sont victimes d´autres personnes, qui ont l´intention de leur causer de la mort.

## Résultats

Entre 1980 et 2014, 611 cas d´intoxications criminelles ont été recensés par le Centre Antipoison et de Pharmacovigilance du Maroc, avec une moyenne de 18 cas par an. Le sexe masculin représentait 55,9% des cas, avec un sexe-ratio (M/F) de 1,3. L´âge moyen des victimes empoisonnées était de 26,4±14,3 ans, avec des extrêmes allant de 5 à 82 ans. La classe d´âge 20-50 ans était particulièrement concernée, avec 58,3%. La majorité (89,4%) des accidents déclarés ont été survenus en milieu urbain. Plus de la moitié des cas ont eu lieu à domicile, soit 54,9% et 25,1% dans un lieu public.

Suivant les données recensées, 149 cas d´empoisonnements collectifs, soit 24,4% ont été enregistrés, avec une exposition répétée pour 13 cas (Tableau 1). D´après les résultats, les produits les plus fréquemment utilisés étaient les pesticides et les produits agricoles, avec 19,1% des cas, suivis par les plantes, avec 19% des cas. L´utilisation des venins d´animaux a été signalée dans 1,8% des cas enregistrés (Tableau 2).

**Tableau 1:** principales caractéristiques de l’empoisonnement criminel au Maroc, CAPM, 1980-2014

Caractéristiques	Nombre de cas (%)	Evolution		Létalité (%)
		Favorable	Décès	
**Sexe**				
Masculin	334 (55,9)	224	20	8,2
Féminin	263 (44,1)	178	7	3,8
n	597 (100,0)	402	27	6,3
**Groupes d'âge**				
Enfant	148 (28,6)	85	4	4,5
Adolescent	82 (15,8)	53	2	3,6
Adulte	285 (55,0)	172	20	9,8
Personne âgée	3 (0,6)	1	-	-
n	518 (100,0)	311	26	7,7
**Milieu**				
Urbain	438 (89,4)	312	18	5,5
Rural	52 (10,6)	37	1	2,6
n	490 (100,0)	349	19	5,2
**Lieu d'intoxication**				
Domicile	247 (54,9)	156	10	6,0
Public	113 (25,1)	91	2	2,1
Ecole	69 (15,3)	67	-	-
Travail	21 (4,7)	14	2	12,5
n	450 (100,0)	328	14	4,1
**Clinique**				
Asymptomatique	81 (13,3)	54	-	-
Symptomatique	530 (86,7)	359	26	6,7
n	611 (100,0)	413	26	5,9
**Gradation**				
Grade 0	46 (10,1)	39	-	-
Grade 1	56 (12,3)	54	-	-
Grade 2	209 (45,8)	161	-	-
Grade 3	118 (25,9)	95	-	-
Grade 4	27 (5,9)	-	27	100,0
n	456 (100,0)	349	27	7,2

**Tableau 2:** répartition des cas d’intoxications criminelles selon les produits utilisés, CAPM, 1980-2014

Agents en cause	Nombre de cas (%)
Pesticides et produits agricoles	109 (19,1)
Plantes	108 (19,0)
Produits industriels	104 (18,3)
Produits gazeux	102 (17,9)
Médicaments	90 (15,8)
Aliments	46 (8,1)
Animaux	10 (1,8)
n	569 (100,0)

Selon le système ou l´organe concerné, les troubles gastro-intestinaux et les troubles du système nerveux central et périphérique étaient les signes cliniques les plus rencontrés, avec respectivement 35,9% et 32,2% des cas. La majorité des cas, soit 86,7% ont été symptomatiques (Tableau 3). L´administration des produits a été effectuée par voie orale dans 72,9% des cas et par inhalation dans 23,8% des cas.

**Tableau 3:** répartition des cas selon le système ou l’organe concerné, CAPM, 1980-2014

Système ou organe concerné	Nombre de cas (%)
Troubles gastro-intestinaux	134 (35,9)
Troubles du système nerveux central et périphérique	120 (32,2)
Troubles psychiatriques	25 (6,7)
Troubles de la fréquence et du rythme cardiaque	25 (6,7)
Affections de l'appareil respiratoire	21 (5,6)
Altération de l'état général	21 (5,6)
Affections du système cardio-vasculaire général	14 (3,8)
Autres	13 (3,5)
n	373 (100,0)

Sur les 440 cas pour lesquels l´évolution est connue, 27 sont décédés, ce qui donne une létalité de 6,1%, avec une létalité spécifique au sexe masculin de 8,2%. Les autres cas ont survécu avec ou sans séquelles. La région de Souss-Massa-Drâa a notifié 8 décès, avec une létalité spécifique de 17,8%. Pour avoir une idée générale sur l´état de santé de la population, on a calculé les principaux indicateurs de santé liés aux intoxications criminelles déclarées au CAPM entre 1980 et 2014.

D´après les résultats, l´incidence annuelle de l´empoisonnement criminel était maximale au niveau de la région de Rabat-Salé-Zemmour-Zaer (1,25 cas pour 1000000 habitants/an) et la région de Tanger-Tétouan (1,09 cas pour 1000000 habitants/an), avec une mortalité annuelle maximale de 0,07 décès pour 1000000 habitants/an dans la région de Rabat-Salé-Zemmour-Zaer (Tableau 4).

**Tableau 4:** indicateurs annuels de santé liés aux intoxications criminelles par région au Maroc, CAPM, 1980-2014

Régions	Nombre de cas	Nombre de décès	Létalité spécifique%	Taux d'incidence*	Taux de mortalité*
Oued Ed-Dahab-Lagouira	1	-	-	0,10	-
Laâ youne-Boujdour-Sakia El Hamra	4	-	-	0,32	-
Guelmim-Es Semara	7	-	-	0,36	-
Souss-Massa-Drâa	45	8	17,8	0,36	0,06
Gharb-Chrarda-Béni Hssen	24	2	8,3	0,34	0,03
Chaouia-Ouardigha	13	-	-	0,21	-
Marrakech-Tensift-Al Haouz	29	-	-	0,24	-
Oriental	45	1	2,2	0,63	0,01
Grand Casablanca	102	3	2,9	0,74	0,02
Rabat-Salé-Zemmour-Zaer	123	7	5,7	1,25	0,07
Doukkala-Abda	16	-	-	0,21	-
Tadla-Azilal	22	-	-	0,41	-
Meknès-Tafilalet	44	1	2,3	0,55	0,01
Fès-Boulemane	17	-	-	0,27	-
Taza-Al Hoceima-Taounate	9	1	11,1	0,13	0,02
Tanger-Tétouan	107	3	2,8	1,09	0,03
n (total)	608	26	4,28	0,52	0,02

* pour 1 000 000 habitants/an

## Discussion

Les intoxications sont une cause fréquente d´admission aux urgences et en réanimation à travers le monde. En 1999, l´OMS a défini une intoxication comme étant une lésion cellulaire ou tissulaire, un trouble fonctionnel ou un décès causé par l´inhalation, l´ingestion, l´injection ou l´absorption d´une substance toxique. Durant la période de l´étude, 611 cas d´intoxications criminelles ont été déclarés au Centre Antipoison et de Pharmacovigilance du Maroc. Ce chiffre est probablement sous-estimé, en raison de la sous notification des cas d´intoxications, en particulier les cas criminels.

Les données ont montré que la majorité des cas était des adultes (55%) et des enfants de 5 à 14 ans (28,6%). Par ailleurs, d´autres travaux menés aux Etats-Unis et en Italie ont montré des fréquences élevées d´intoxications chez les enfants, dont l´âge est compris entre 0 et 4 ans (respectivement 48,5% et 37%) [6,7].

Nos résultats ont montré que le sexe masculin est le plus exposé au risque d´intoxications criminelles, avec un sexe-ratio (M/F) de 1,3, ceci peut être expliqué par le fait que le poison était, depuis les temps les plus reculés, l´arme favorite des femmes, qui veulent mettre fin à la vie de leurs conjoints. Parmi ces actes, on cite l´exemple célèbre de Marie Besnard et l´affaire Lafarge. Cependant, les femmes restent plus exposées au risque d´intoxications, ce qui concorde avec les résultats d´autres études menées en Belgique et au Royaume-Uni [8,9]. Cependant, en Australie, les hommes ont été les plus exposés au risque d´empoisonnement [10].

D´après les données de cette étude, les intoxications criminelles étaient survenues en milieu urbain dans 89,4% des cas, ceci peut être expliqué par la sous notification des zones rurales. Les pesticides, les plantes et les produits industriels étaient les produits les plus utilisés, avec respectivement 19,1%, 19% et 18,3% des cas.

Dans plusieurs pays, les produits les plus couramment utilisés dans le cas des intoxications intentionnelles sont les pesticides, ce qui s´accorde avec nos résultats [11-13]. Ce type d´intoxication peut engendrer de graves effets sur les intoxiqués pouvant conduire à la mort [14]. Par ailleurs, les intoxications par les plantes sont accidentelles chez les enfants et suicidaires chez les adultes, et elles représentent de faibles fréquences à travers le monde [15-17]. Le degré de sévérité de l´ingestion des plantes dépend de la nature de la plante et parfois même de la partie utilisée et la dose administrée [18]. Les plantes ont été utilisées depuis l´antiquité et l´histoire du poison ne cesse d´enrichir la littérature sur les principes actifs utilisés à des fins criminelles au fil du temps. Un simple exemple, les amandes de noyaux de pêches étaient broyées pour obtenir du cyanure. Ce poison était mélangé avec des aliments et provoquait des signes différents selon la dose prise. Une forte quantité conduisait à la mort [19].

Nos résultats ont montré aussi l´utilisation des venins d´animaux dans des actes malveillants à visée criminelle [20]. D´après notre étude, toute une série de symptômes gastro-intestinaux, neurologiques et respiratoires étaient observés, ceci est dû à la diversité des produits toxiques utilisés dans ce type d´intoxication. Les intoxications criminelles étaient responsables de 27 décès, ce qui représente une létalité de 6,1%. Ce taux est probablement sous-estimé vu que la confusion avec les cas liés aux circonstances suicidaires ou accidentelles est très probable.

## Conclusion

L´empoisonnement criminel, très peu documenté dans la littérature, constitue un problème non négligeable. L´administration de substances à l´insu de la victime à des fins criminelles, qui peut être la cause d´une intoxication involontaire grave impose une sensibilisation des médecins confrontés aux problèmes de diagnostic et de prise en charge des victimes. Une mise en œuvre de stratégies, ayant pour objectifs de limiter la vente libre de certains produits tels que les pesticides et connaître les causes réelles de ce type d´intoxication, est très recommandée.

### Etat des connaissances sur le sujet

- L´intoxication criminelle est peu documentée dans la littérature;
- Les résultats publiés concernant juste la fréquence de ce type d´intoxication par rapport aux intoxications intentionnelles.

### Contribution de notre étude à la connaissance

- Cette étude présente le profil épidémiologique des intoxications criminelles déclarées au CAPM;
- Elle présente les produits les plus fréquemment utilisés dans les actes malveillants à visée criminelle et donne le profil des victimes.

## Remerciements

Ce travail est mené dans le cadre du projet: PPR-B-MOKHTARI-FS-UIT-Kénitra.

## References

[1] Smith KR, Corvalan CF, Kjellstrom T (1999). How much global ill health is attributable to environmental factors. Epidemiology.

[2] Bédry R, Nouts C, Guisset O (2006). International prospective study of practice into the ventilatory management of the poisoned patients. Clinical Toxicology.

[3] Centre Suisse d'Information Toxicologique (2018). Rapport annuel du centre suisse d'information toxicologique, 2018.

[4] Chaoui H, Rhalem N, Badri M, Ouammi L, Soulaymani-Bencheikh R (2014). Rapport général 2014 de toxicovigilance. Toxicologie Maroc.

[5] Persson HE, Sjöberg GK, Haines JA, Pronczuk de Garbino J (1998). Poisoning severity score: grading of acute poisoning. J Toxicol Clin Toxicol.

[6] Bronstein AC, Spyker DA, Cantilena LR, Green JL, Rumack BH, Dart RC (2011). 2010 annual report of the American association of poison control centers' national poison data system (NPDS): 28^th^ annual report. Clin Toxicol (Phila).

[7] Mucci N, Alessi B, Binetti R, Magliocchi M (2006). Profilo delle intossicazioni acute in Italia: analisi dei dati registrati dai centri antiveleni. Ann Ist Super Sanità.

[8] (2012). Centre Antipoison Belgique. Rapport d'activité 2012.

[9] Jackson G, Good AM, Bateman DN (2010). National poisons information service annual report 2009/2010 and five-year review. Health Protection Agency London.

[10] Huynh A, Cairns R, Brown J, Lynch A, Robinson J, Wylie C (2018). Patterns of poisoning exposure at different ages: the 2015 annual report of the Australian Poisons Information Centres. Med J Aust.

[11] Gunnell D, Eddleston M (2003). Suicide by intentional ingestion of pesticides: a continuing tragedy in developing countries. Int J Epidemiol.

[12] Van Der Hoek W, Konradsen F (2005). Risk factors for acute pesticide poisoning in Sri Lanka. Trop Med Int Health.

[13] Mathieu-Nolf M (2005). Rapport annuel d'activité du centre antipoison et de toxicovigilance de Lille.

[14] Derkaoui A, Elbouazzaoui A, Elhouari N, Achour S, Labib S, Sbai H (2011). Intoxication aiguë sévère par les pesticides organophosphorés: à propos de 28 cas. Pan African Medical Journal.

[15] Bagnis CI, Deray G, Baumelou A, Le Quintrec M, Vanherweghem JL (2004). Herds and the kidney. American Journal of Kidney Diseases.

[16] Flesch F (2005). Intoxications d'origine végétale. EMC-Médecine.

[17] Nisse P (2003). Intoxications par les végétaux: plantes et baies.

[18] Dauvin E (2009). Intoxication par les plantes: site internet d´aide à la reconnaissance de la plante et à la prise en charge de l'intoxiqué. Sciences du Vivant.

[19] Flahaut M (2015). Le cyanure dans l'histoire et intoxications actuelles. Médecine Humaine et Pathologie, Thèse de doctorat.

[20] Mebs D (2006). Animaux venimeux et vénéneux. Ed Tec et Doc.

